# Immunotherapy-Induced Eosinophilic Tracheitis

**DOI:** 10.7759/cureus.24130

**Published:** 2022-04-13

**Authors:** Eduardo Tellez-Garcia, Alejandra Valdivia Padilla, Horiana Grosu

**Affiliations:** 1 Internal Medicine, Instituto Tecnologico y de Estudios Superiores de Monterrey, Monterrey, MEX; 2 Pulmonary Medicine, Monroe Dunaway (MD) Anderson Cancer Center, Houston, USA

**Keywords:** nivolumab-related adverse events, melanoma, chronic cough, tracheitis, immunotherapy

## Abstract

The present report describes the case of a 27-year-old male with a longstanding diagnosis of melanoma with metastatic disease treated with nivolumab, an immune checkpoint inhibitor. He presented with a chronic cough of unknown etiology, not responsive to standard of care treatment with cough suppressants. In view of this, a bronchoscopy was done, and biopsies were taken. The patient was diagnosed with eosinophilic tracheitis secondary to nivolumab. His symptoms improved with steroids and cessation of the immune checkpoint inhibitor. Prescribing physicians should be aware of these rare and potentially life-threatening toxic effects.

## Introduction

Immunotherapy has established itself as a new standard of care in the management of many cancers, as evidence has shown significant improvement in survival and disease-free period [1,2]. Nivolumab is an immunotherapy agent that belongs to the group of immune checkpoint inhibitors. However, these new medications are not exempt from diverse undesired effects. The adverse effects elicited by eosinophilic infiltration in respiratory and non-respiratory tissues secondary to nivolumab are beginning to be more recognized. Furthermore, specific adverse effects in the respiratory tract, such as pneumonitis [3], bronchiolitis [4], and rhinosinusitis [5], have been reported. However, to the best of our knowledge, no other case of eosinophilic tracheitis without concomitant pneumonitis induced by nivolumab has been published before.

## Case presentation

**Figure 1:**
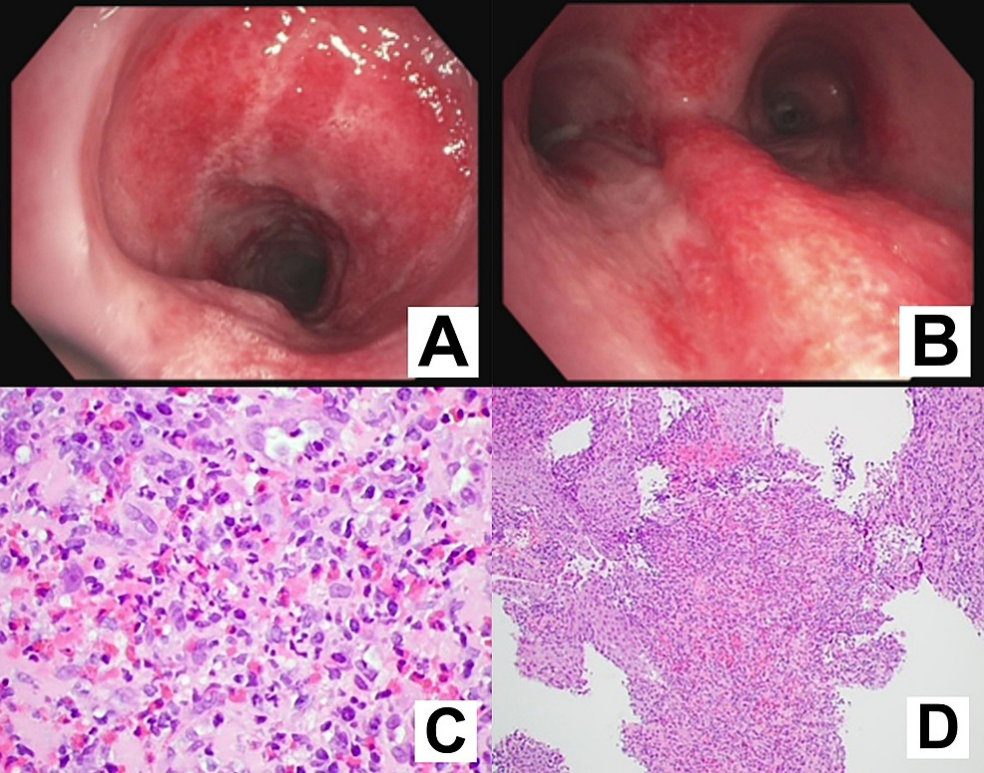
A: Bronchoscopic view of the subglottic space with significant erythema and a nodular appearance. B: Bronchoscopic view of the distal trachea and main carina with significant erythema and a nodular appearance. C: Endobronchial biopsy showing fragments of the tracheal wall infiltrated by numerous inflammatory cells (H&E: ×10). D: High-power magnification showing that the majority of the inflammatory cells are eosinophils (H&E: ×60).

A 27-year-old male was diagnosed with metastatic melanoma in September 2018 and started on treatment with immune checkpoint inhibitors ipilimumab and nivolumab. His treatment was complicated by immunotherapy-induced hepatitis, which required holding of treatment and a prolonged course of steroids. His immunotherapy-induced hepatitis improved significantly, but because of disease progression, he was started on encorafenib and binimetinib and challenged with nivolumab. Later on, he devolved recurrent bloody diarrhea; thus, an abdominal computer tomography (CT) scan was obtained, which showed abdominal wall thickening suggestive of colitis. A colonoscopy was performed, and biopsies were taken. Pathology was consistent with immune-induced colitis, once more requiring a treatment hold and steroids. His condition improved, and he was again rechallenged with nivolumab. The possible risks of this were explained thoroughly to the patient, and he consented to it. Some months after, while on nivolumab, he developed a cough; however, imaging showed no evidence of pneumonitis, and pulmonary function test results were normal. A few months into his treatment with nivolumab, his cough worsened and was accompanied by low-grade fever. Antibiotic treatment was administered, with only a slight clinical improvement. Additionally, a chest CT scan demonstrated a right lower lobe lung abscess. In view of this, a bronchoscopy with bronchoalveolar lavage was performed. The bronchoscopy showed significant erythema and a nodular appearance in the subglottic space, trachea, and main carina (Figure 1A, 1B). Biopsy of the trachea showed the formation of granulation tissue with florid eosinophilic inflammation (Figure 1D, 1E). The patient was diagnosed with immunotherapy-induced tracheitis, and immunotherapy was once more placed on hold and steroids initiated, with significant improvement in symptoms (Figure 1C). Unfortunately, the patient’s cancer progressed, and he died shortly thereafter.

## Discussion

Nivolumab is a human monoclonal antibody that blocks the programmed death-1 receptor (PD-1 or CD279) [6]. It belongs to a group of immunotherapy drugs known as immune checkpoint inhibitors and is used to treat lung cancer, melanoma, renal cell carcinoma, squamous cell carcinoma, mesothelioma, urothelial carcinoma, and other cancers [6-8]. Treatment with nivolumab can generate adverse effects, such as fatigue, decreased appetite, nausea and vomiting, cough, dyspnea, and rash [6]; moreover, several cases of rarer adverse effects have been published, such as vitiligo [9], thyroiditis [10], myositis [11], polyarthritis [12], vasculitis [13], and cholangitis [14], among others. Specific adverse effects on the respiratory system have also been reported, including pneumonitis [3] and rhinosinusitis [5]. Similar to our case, some studies have shown eosinophilic infiltration of the affected organ [5,14]. The specific mechanism through which eosinophils get recruited to the airway is yet to be fully elucidated, although evidence has shown that antibodies against PD-1 activate Th2 cells, which secrete IL-5 that attracts eosinophils to tissues [15]. To the best of our knowledge, no other case of eosinophilic tracheitis induced by nivolumab in the absence of pneumonitis has been published before. The European Society for Medical Oncology (ESMO) provides guidelines for the management of the toxic effects of immunotherapy, but no specific treatment for immunotherapy-induced tracheitis is provided [16].

## Conclusions

Immunotherapy is an exciting but still vastly unknown growing field. Future studies will completely establish its role in the management of patients with cancer. Physicians who prescribe nivolumab or attend patients on treatment with it should be aware of the potential adverse effect we have exposed in this case report, especially in patients who develop chronic cough without a clear explanation. Further studies are required to increase our understanding of the mechanism behind the toxic effects of immunotherapy so that we can improve their management.
